# Qualitative investigation of the factors that generate ambivalent feelings in women who give birth after receiving negative results from non-invasive prenatal testing

**DOI:** 10.1186/s12884-020-2763-z

**Published:** 2020-02-17

**Authors:** Junko Yotsumoto, Akihiko Sekizawa, Satomi Inoue, Nobuhiro Suzumori, Osamu Samura, Takahiro Yamada, Kiyonori Miura, Hideaki Masuzaki, Hideaki Sawai, Jun Murotsuki, Haruka Hamanoue, Yoshimasa Kamei, Toshiaki Endo, Akimune Fukushima, Yukiko Katagiri, Naoki Takeshita, Masaki Ogawa, Haruki Nishizawa, Yoko Okamoto, Shinya Tairaku, Takashi Kaji, Kazuhisa Maeda, Keiichi Matsubara, Masanobu Ogawa, Hisao Osada, Takashi Ohba, Yukie Kawano, Aiko Sasaki, Haruhiko Sago, Japan NIPT Consortium, Seiji Wada, Miyuki Nishiyama, Akira Namba, Hisao Osada, Yasuyo Kasai, Atsushi Watanabe, Kazufumi Haino, Naoki Hamajima, Takeshi Kanagawa, Hiroaki Nakamura, Jun Yoshimatsu, Katsuhiko Naruse, Hisashi Masuyama, Maki Hyodo, Rina Akaishi, Takashi Kojima, Yuka Shibata, Nahoko Shirato, Keiko Miyagami, Tatsuko Hirose, Atsuko Saito, Yuri Hasegawa, Shoko Miura, Noriko Sasaki, Mako Ueda, Mariko Ushioda, Chiho Okada, Hiroyuki Tanaka, Mina Morii-Kashima, Kyoko Kumagai, Eri Takeda, Kumiko Oseto, Wakana Abe, Kimiko Enomoto, Yoshinobu Sugo, Mari Shinoda, Kitagawa Michihiro

**Affiliations:** 1 Department of Genetic Counseling, Graduate School of Health and Welfare Sciences, International University of Health and Welfare, 4-1-26 Minato-ku, Tokyo, 107-8402 Japan; 2 Department of Obstetrics and Gynecology, Showa University School of Medicine, Tokyo, Japan; 3 Medical Genetics Center, National Hospital Organization Tokyo Medical Center, Tokyo, Japan; 4 Division of Clinical and Molecular Genetics, Department of Obstetrics and Gynecology, Nagoya City University Graduate School of Medical Sciences, Nagoya, Japan; 5 Department of Obstetrics and Gynecology, The Jikei University School of Medicine, Tokyo, Japan; 6 Clinical Genetics Unit, Kyoto University Hospital, Kyoto, Japan; 7 Department of Obstetrics and Gynecology, Nagasaki University Graduate School of Biomedical Sciences, Nagasaki, Japan; 8 Department of Obstetrics and Gynecology, Hyogo College of Medicine, Nishinomiya, Japan; 9 Department of Maternal and Fetal Medicine, Tohoku University Graduate School of Medicine, Miyagi-Children’s Hospital, Sendai, Japan; 10 Department of Clinical Genetics, Yokohama City University Hospital, Yokohama, Japan; 11 Department of Obstetrics and Gynecology, Saitama Medical University Hospital, Saitama, Japan; 12 Department of Obstetrics and Gynecology, Sapporo Medical University School of Medicine, Sapporo, Japan; 13 Department of Clinical Genetics, School of Medicine, Iwate Medical University, Morioka, Japan; 14 Department of Obstetrics and Gynecology, Faculty of Medicine, Toho University, Tokyo, Japan; 15 Perinatal Medical Center, Tokyo Women’s Medical University Hospital, Tokyo, Japan; 16 Department of Obstetrics and Gynecology, Fujita Health University, Aichi, Japan; 17 Department of Obstetrics, Osaka Medical Center and Research Institute for Maternal and Child Health, Osaka, Japan; 18 Department of Obstetrics and Gynecology, Kobe University Graduate School of Medicine, Kobe, Japan; 19 Department of Obstetrics and Gynecology, The University of Tokushima Faculty of Medicine, Tokushima, Japan; 20 Department of Obstetrics and Gynecology, Shikoku Medical Center for Children and Adults, Kagawa, Japan; 21 Department of Obstetrics and Gynecology, Ehime University School of Medicine, Ehime, Japan; 22 Department of Obstetrics and Gynecology, Clinical Research Institute, National Hospital Organization Kyushu Medical Center, Fukuoka, Japan; 23 Department of Maternal-fetal Medicine, Chiba University Hospital, Chiba, Japan; 24 Department of Obstetrics and Gynecology, Kumamoto University, Kumamoto, Japan; 25 Department of Molecular Pathology, Faculty of Medicine, Oita University, Oita, Japan; 26 Center for Maternal-Fetal, Neonatal and Reproductive Medicine, National Center for Child Health and Development, Tokyo, Japan

**Keywords:** Ambivalence, Genetic counseling, NIPT, Anticipatory anxiety, Content analysis

## Abstract

**Background:**

Women who receive negative results from non-invasive prenatal genetic testing (NIPT) may find that they later have mixed or ambivalent feelings, for example, feelings of accepting NIPT and regretting undergoing the test. This study aimed to investigate the factors generating ambivalent feelings among women who gave birth after having received negative results from NIPT.

**Methods:**

A questionnaire was sent to women who received a negative NIPT result, and a contents analysis was conducted focusing on ambivalent expressions for those 1562 women who responded the questionnaire. The qualitative data gathered from the questionnaire were analyzed using the N-Vivo software package.

**Results:**

Environmental factors, genetic counseling-related factors, and increased anticipatory anxiety, affected the feeling of ambivalence among pregnant women. Furthermore, pregnant women desired more information regarding the detailed prognosis for individuals with Down syndrome and living with them and/or termination, assuming the possibility that they were positive.

**Conclusions:**

Three major interrelated factors affected the feeling of ambivalence in women. Highlighting and discussing such factors during genetic counseling may resolve some of these ambivalences, thereby enhancing the quality of decisions made by pregnant women.

## Background

A prenatal test for fetal abnormalities may cause maternal anxiety [1, 2]. Pregnant women who made an uninformed choice for non-invasive prenatal genetic testing (NIPT) felt deeper decisional regret associated with prolonged anxiety [3, 4].

Women typically have two conflicting thoughts at the early stage of pregnancy: the desire to know about the neonate via ultrasound examination and the reluctance to receive negative news. Many women reportedly take the NIPT to relieve their ambivalence regarding negative results [5]. Furthermore, certain women feel like their pregnancy is provisional or temporary until they learn about their NIPT results, i.e., although they may feel physical changes, they may not acknowledge their pregnancy until they see their results [6]. Ambivalence has been identified among the attitudes of the general public regarding prenatal testing; however, the origins of the ambivalence remain unclear [7, 8]. Lewis et al. reported on the ambivalence of the women taking the NIPT had ambivalent feelings, e.g., desire for the information about the baby by the test and additional anxiety whilst waiting, concerns around “over-testing” and “pressure by the health professionals” [9].. Ambivalence can be defined as the “simultaneous existence of positive and negative evaluations of an attitude” [10].

In our previous study, we conducted an “awareness survey of NIPT in Japan”, and reported that women with negative results had a higher NIPT rating than women with positive results, but with respect to the ethical aspects of NIPT, women with negative results responded unclearly [11]. In the presence of negative NIPT results, diseases other than the three chromosomal aneuploidies may be identified during later stages of life [12] then they are expected to have mixed feelings about the NIPT.

Based on these findings, we thought that even women with negative test results could have ambivalent feelings later. This study aimed to investigate factors affecting ambivalent feelings in pregnant women after undergoing NIPT.

## Methods

### Design

The present study is based on a qualitative research methodology, involving content analysis using the free-form description of the first year after questionnaire answered by women who received negative results for NIPT.

### Research procedure

**Fig. 1:**
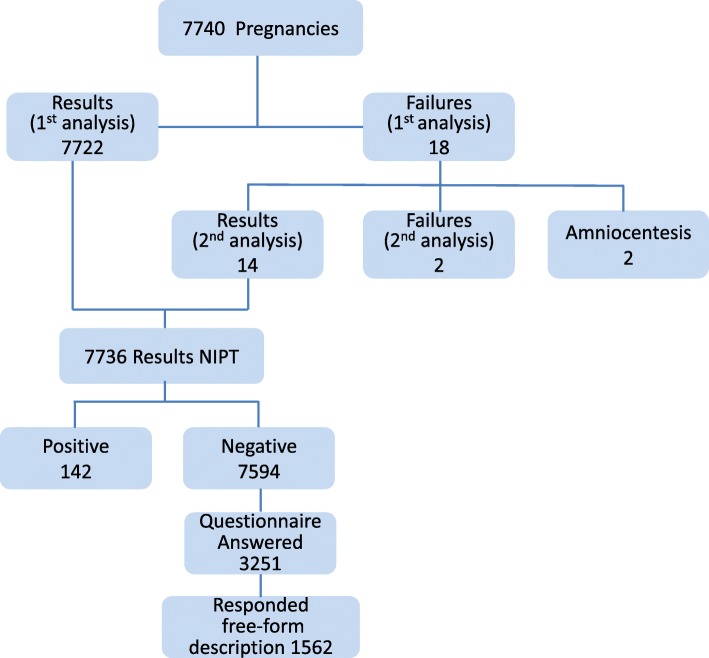
Flowchart of the respondent selection

All pregnant women who took the NIPT were asked to participate in a questionnaire-based survey conducted by the Japan NIPT Consortium. The survey was in two parts: (i) pre-and immediately after the NIPT test, from April 2013 to March 2014 and (ii) a year- after the NIPT test, from April 2014 to March 2015. We have already reported on part (i) [11], and the present study is for part (ii). A year-after research was conducted by a mail-in survey to determine whether the assessment of the NIPT and genetic counseling was changed after the test and 1 year after. The contents of the questionnaire consisted of a one-year-after evaluation and free description for NIPT and genetic counseling, and the question of the free description part was an open-ended question, such as, “Please show your opinion or feedback of the NIPT.” In the present study, we conducted content analysis for 1562 respondents (20.6%) who provided the free-form description to the open-ended question (Fig. 1).

### Participants

Pregnant women who were identified as being at an increased risk of chromosomal disorders due to advanced maternal age, the results of ultrasound marker or maternal serum marker tests, or a family history of chromosomal abnormalities or those who had a high risk of being a translocation carrier, were eligible for NIPT. In most cases, the indication for NIPT was advanced maternal age (96.5%). In addition, 14.4% of the women underwent NIPT based on a recommendation from their family. Because the responses in the first year after the questionnaire were anonymous, and it was impossible to obtain the background data, the personal background of the respondents was unknown; however, the available necessary background information of the respondents who underwent NIPT was shown in the previous study [11] of 7740 women, whereas the present study was limited to those who received negative NIPT results among the 7740 women, and therefore, the background data were not same.

### Data analysis

The questionnaires were subsequently mailed to a third-party NIPT data center for data entry. Qualitative methods are required in situations warranting detailed analysis and an understanding of the process to determine the nature of the issue being investigated. We undertook a combined content analysis (quantitative and qualitative) via text mining approaches, using N-Vivo Qualitative software for the free-form description part of the questionnaire focusing on ambivalent expressions. The advantage of computer-assisted content analysis of textual data is the coding reliability that helps to generate comparable results [13]. The text in the free-form description part was entered into the computer and used as input for the N-vivo software. N-vivo was used for line-by-line coding of the texts, to identify emerging themes. Furthermore, all the text was read, and the coding process was discussed with two researchers. The sub-categories extracted by N-vivo were assessed by two reviewers. The code of those lower categories determined after the evaluation was extracted inductively into the above category by two reviewers. The coding process is shown below.

- Step 1 Line-by-line coding with N-vivo, using an inductive approach.
- Step 2 Thematic grouping. The transversal analysis showed new themes by two researchers.
- Step 3 Creating higher-level categories and evaluating relationships.
- Step 4 Reviewing these analyses. Thematic stabilization. All authors.

### Ethics

To conduct this study, the participating centers obtained approval from their respective ethics committees.

## Results

**Fig. 2:**
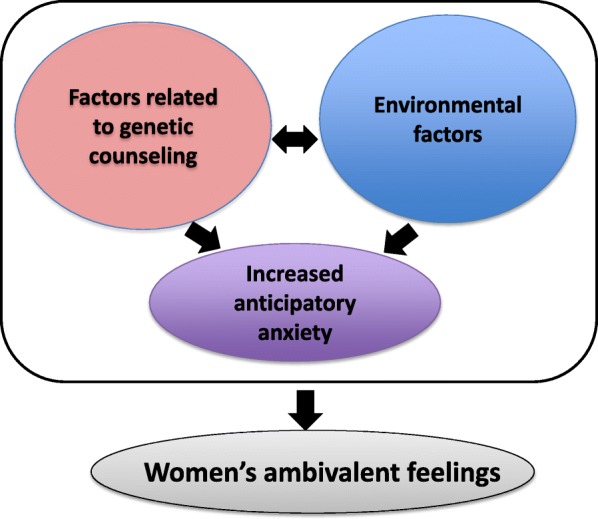
The relationship between women’s ambivalent emotions and their factors

We identified three primary categories generating feelings of ambivalence among women: (1) factors related to genetic counseling; (2) environmental factors, and (3) increased anticipatory anxiety. Furthermore, the former two factors caused the anticipatory anxiety (Table.1, Fig.2).

**Table 1:** Factors associated with ambivalence

Primary category	Subcategory	Mid-level category	Lowest category
Factors related to genetic counseling (*n* = 191)			
Lack of information (159)			
Inadequate support by medical staff up to taking NIPT (69)			
Lack of neutrality in genetic counseling (*n* = 11)			
Lack of NIPT information from family physician before genetic counseling (*n* = 26)			
Family physician’s response (*n* = 33)			
Lack of information in case of positive result (*n* = 91)			
if the pregnancy was terminated (*n* = 14)			
if the pregnancy was continued (*n* = 51)			
follow-up support to positive result (*n* = 55)			
Lack of psychological care adapted to individual needs (*n* = 48)			
Lack of psychological care at genetic counseling (*n* = 35)			
Lack of genetic counseling at the appropriate moment (*n* = 17)			
Environmental factors (82)			
Lack of awareness and education about diversity (*n* = 30)			
Insecurities on raising the child (*n* = 63)			
Insecurities for the future (*n* = 26)			
After the parents’ death (*n* = 20)			
Impact on the siblings (*n* = 17)			
Vague insecurity (*n* = 20)			
Insecurities for the child-rearing environment (*n* = 18)			
Familiarity with challenges of living with disabled person (*n* = 9)			
Lack of social support systems for people with disabilities (*n* = 23)			
Increased anticipatory anxiety (*n* = 56)			
Time related anxiety (*n* = 47)			
Anxiety about test accuracy (*n* = 9)			

Footnote: *n* Number of occurrences of each category. Categories may partially overlap

### Factors related to genetic counseling

This category ***Factors related to genetic counseling*** comprised two subcategories: “Lack of information” and” Lack of psychological care adapted to individual needs.” “Lack of information” further comprised two mid-level categories: “Inadequate support by medical staff up to taking NIPT” and “Lack of information in case of positive result.” “Lack of psychological care adapted to individual needs comprised “Lack of psychological care at genetic counseling,” and “Lack of genetic counseling at the appropriate moment.”

Regarding “Lack of information,” “support by medical staff up to taking NIPT” referred to the lack of support from the family physician during NIPT. Many family physicians were not only not helpful in referring their patient for genetic counseling, but they also themselves were unaware of it. Owing to the limited number of NIPT facilities for pregnant women in Japan, some respondents also reported receiving a reproachful response from their family physician upon requesting information regarding NIPT or referrals.

Furthermore, certain women were criticized for taking the NIPT during genetic counseling, and many women wished that genetic counseling would be performed assuming that NIPT could give positive results regarding “Lack of information in case of positive result.” They wished to receive detailed information regarding the methods of terminating their pregnancy as one of the alternatives in case of chromosomal abnormalities in the fetus.

*I don't think it hurts to take some time to explain about the options for getting an artificial termination of pregnancy in the case of a positive result. There is this idea that an abortion=bad, but if the chance that you can love your child unconditionally is 0%, then I believe that it is an important option, even though it's not easy to discuss...*

*(if the pregnancy was terminated)*

However, some women who considered continuing their pregnancy despite receiving positive results upon NIPT were seeking information regarding the exact quality of life for individuals living with such illnesses and the social resources available for them.

*What I really want to see added is what to do if the results do come out positive. I want to hear during the counseling session the exact types of social support that I could get if my child is born with Down syndrome or some other illness to help me live with and raise the child with the illness, for example at school, daycare, at home, names of organizations or municipal centers where I can get support.*

*(if the pregnancy was continued)*

These data indicated that respondents were seeking information during genetic counseling that would support the decision to continue or terminate their pregnancy. These women desired information regarding the daily lives of children with disabilities and other related materials to guide their decision of whether they could raise a child with disabilities; furthermore, they desired follow-up support for women reporting with positive results in the NIPT.

### Environmental factors

***Environmental factors*** included the comfort levels of women for raising a child with disabilities; this aspect comprised three subcategories: “Lack of awareness and education about diversity,” “Insecurities on raising the child,” and “Lack of social support systems for people with disabilities. “Insecurities on raising the child” comprised “Insecurities for the future,” “Vague insecurity,” “Insecurities for the child-rearing environment,” and “Familiarity with challenges of living with a disabled person.”

*• • • The reason why many people have the image of prenatal testing as an “immoral thing” in Japan may come from views about ethics on this subject in Japan, or prejudice and closed-mindedness against people with Down syndrome and other chromosomal abnormalities. I just sometimes wish that those who are debating and giving their input on ethics would also take part in discussing how to change Japan into a more livable society for children with Down syndrome and their families.*

*(Lack of awareness and education about diversity)*

*I want to give birth even if child with Down syndrome, if possible ... The word “life sorting” is not good, but we feel that social systems are forced to do so. I think we must create a welfare, education and social system that everyone can give birth and raise without worrying, even if a child has a disability.*

*(Lack of social support systems for people with disabilities)*

Regarding “Insecurities on raising the child”, older parents had a sense of responsibility to raise the child; they were concerned regarding the life of the child after their death owing to their age at pregnancy, and they were concerned regarding burdening the siblings of the child and, thus, had “Insecurities for the future.”

Furthermore, certain respondents also exhibited “Vague insecurity” because raising a disabled child is unfathomable and difficult to imagine, owing to the lack of experience. Others, however, already had “Familiarity with challenges of living with a disabled person,” because they knew somebody or had family members with disabilities, thereby fostering the understanding of the merits and demerits and having discerned that it would be challenging. They also faced “Insecurities for the child-rearing environment” owing to their concern regarding an inadequate financial support or the social environment for raising a child with disabilities.

*As an actual mother of a child with chromosomal abnormalities • • •I’m glad that I had my baby, but raising my child is full of challenges and would be absolutely impossible without the cooperation of people around me, so it's not something that can be glossed over. I got tested for my second pregnancy, but I don't think I would've had the child if the results were positive.*

*(Familiarity with challenges of living with a person with disabilities).*

### Increased anticipatory anxiety

***Increased anticipatory anxiety*** included the two subcategories” Time-related anxiety” and “Anxiety about test accuracy.” Among the pregnant women who opted for NIPT, there were some who were so anxious during the 2 weeks until they got the results that they could not sleep until finding out that their results were not positive. Also, knowing that test accuracy of NIPT was not 100%, some people were anxious that their result might be a false-negative, and they could not eliminate this anxiety until the birth of the child.

### Components of ambivalence

***Ambivalent feeling*** comprised five subcategories: “Options in the case of a positive result,” “Guilt towards the child,” “Criticisms on NIPT from others,” “Denial of disabled people”, and “How to tell the child.”

**Fig. 3:**
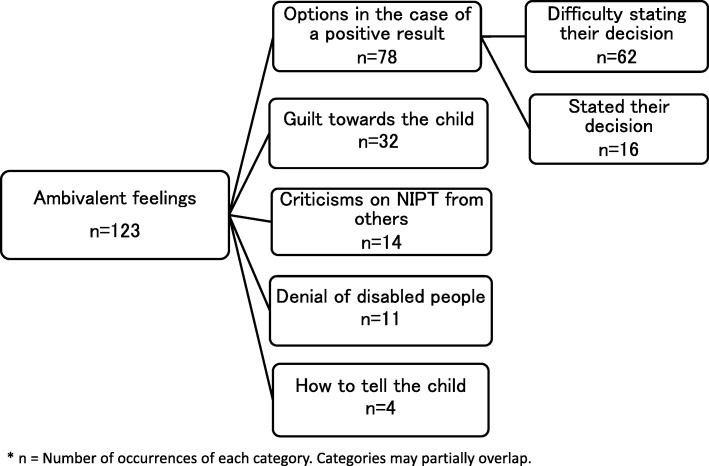
Components of the ambivalence

Some respondents “Stated their decision” with regard to the course of action in case of a positive NIPT result, whereas others expressed “Difficulty stating their decision” regarding continuing or terminating their pregnancy. In both cases, respondents seemed agonized over their decision in the case of positive results (Fig. 3).

*I had decided that if the results came out positive, I would not give birth to the baby. However, even though I now know that the results were negative, I haven't told my own parents that I took the test. At the same time that I feel negative about the idea that we were picking and choosing who to keep if the results were positive, I also blame myself for my lack of confidence in raising the child, even though it is because I have health problems myself.*

*(Stated their decision).*

Some women seemed to have a sense of “Guilt towards the child” after given birth and parenting the child, knowing that they were trying to decide the life of the child.

*I don't think I would have taken the test if I were younger. Some people criticize that people take the test too casually because it places very little stress on the mother or child. But I believe that anybody who chose to take the test did so after putting lots of thought into it. Looking into my child's face, sometimes I remember about when I took the test, and I can't help feeling sorry towards my child. My baby's life is very valuable, and I want to give all the love it deserves.*

*(Guilt towards the child)*

Furthermore, some women could not tell anybody that they took the NIPT owing to social pressure,” Criticisms from others on NIPT”, and others who felt guilty that having “Denial of disabled people” upon having children with disorders, which were screened during NIPT, despite obtaining negative NIPT results.

*When I watch documentaries on TV, etc., and hear that the majority of opinions seem to support that “Whether or not a life should live should not be decided by parents, but should be treated as an independent life” “Even if my child has Down syndrome, I am so happy that they came to this world”. People who, for whatever reason decided not to have the baby, will be burdened with guilt for the rest of their lives. I did test negative, but whenever I look into my child's face and see a person with Down syndrome, I criticize myself because “I decided on her life.”*

*(Denial of disabled people)*

## Discussion

The results of this study also showed that factors related to genetic counseling and environmental factors greatly influence women’s decision-making regarding taking the NIPT. Because of the impact of factors related to adequate information, i.e., ***Factors related to genetic counseling*** before undergoing NIPT and ***Environmental factors***, which describe an inadequate social environment wherein the ability of independent decision making among women is perturbed, ***Anticipatory anxiety*** is increased. The decision-making among pregnant women who receive a prenatal diagnosis is affected not only by individuals close to them, e.g., family members or friends, but also by the opinions of medical staff, the social environment, and uncertainty regarding fetal health conditions [13–16].

Furthermore, the guilt that women experience when deciding to give birth or abort the fetus, or the guilt they experience regarding decision-making related to disabled individuals and contrary sentiments associated with ideas that promote prenatal testing and that having to face this moral opposition induces a state of ***Ambivalence*** among some women even 1 year after taking the NIPT, whenever the unresolved feelings associated with the thought “What if the result had been positive *• • •*” resurfaced in their minds.

There are problems related to genetic counseling about assuming positive NIPT results. Genetic counselors themselves are concerned that talking to parents about Down syndrome during prenatal testing is likely to elicit fear among them. Overly optimistic or negative information from medical staff, who largely influence the parents’ ultimate decision-making upon receiving positive NIPT results can also introduce a bias [17, 18], similar to the genetic counselors themselves who lack the necessary skills and knowledge [3]. Perhaps, this is focused on avoiding any negative effects of providing information while assuming a positive NIPT result on the mental status of pregnant women, who seek emotional relief from undergoing the test. However, many women also seek information regarding the precise living conditions of children with the screened genetic disorders and regarding the types of social resources available to them.

Statistics have revealed that in England, a few more women who received positive NIPT results chose to have an abortion compared to those who tested positive upon amniocentesis [3]. Concurrently, in Japan, the possibility of continuing the pregnancy existed among many women, despite obtaining positive NIPT results. The present results also show that it is important to offer genetic counseling under the assumption of a potentially positive NIPT result.

Among numerous pregnant women, the motivation to undergo prenatal testing stems from their need to “feel relieved [5].” However, among the women who inquire about prenatal diagnosis, many request information regarding not only about the actual test, but also regarding the many anxieties they may have about their children during pregnancy. Pregnant women attended NIPT genetic counseling sessions at a time when they could openly speak and be listened to; hence, it is very important to provide psychological support to pregnant women in addition to information regarding testing methods or disorders that can be screened for during the test [19].

Fears regarding the potential discrimination faced by the child, prejudice, the lack of understanding among family members or other people, and the lack of societal support for raising the child are some of the reasons that motivate women to have a “healthy” child. There is a cultural feature in Japan that is sensitive to the surroundings and is easily affected. Furthermore, the present results suggest that the society is not very open and livable for individuals with disabilities. Simultaneously, numerous individuals strongly felt the need to adapt to the social support systems for people giving birth to children with disabilities. These results suggest a positive and supportive perspective towards disabled individuals, fostering the hope that in the future, diversity will be widely accepted and that society will be welcoming to all children, irrespective of being born with disabilities. Many of the present study subjects were mothers with late-life pregnancies. We believe the potential impacts on family and siblings, societal prejudices on disabilities, and attachment towards the unborn child were weighed after careful consideration and understanding of societal prejudices and the increased chance of having a child with an abnormal chromosomal number owing to their age before choosing to undergo NIPT.

Similar to other types of prenatal testing, we believe that NIPT may also increase the pregnant women’s worries and anxieties about the unborn child. The rate of infertility treatment was high among the women who underwent NIPT (42.2%). Furthermore, women undergoing infertility treatment have strong fears regarding chromosomal abnormalities among their children ([20] McMahon, 2013 #895, [21]); however, informing women that results are usually negative for 98% of older mothers may as well reduce the anxiety among women undergoing infertility treatment [22, 23]. Offering detailed information to pregnant women during genetic counseling sessions to enable its juxtaposition with the mothers’ own experiences and values to make an informed decision whether or not to undergo NIPT lowers the amount of uncertainty, stress, and anxiety they experience during the decision-making [24, 25].

The present study indicate that numerous individuals seek detailed information regarding the survival of children with positive results upon NIPT, including social support services, which indicates the lack of information and its accuracy regarding these disorders. As such, administration of NIPT is accompanied by high levels of stress among mothers, thereby potentially explaining their increased levels of anxiety until receiving the test results or their anxieties regarding the accuracy of the test [26].

Pregnant women taking the NIPT are compelled to gain adequate knowledge and understanding of the NIPT and the disorders screened therewith and to decide between continuing or terminating their pregnancy upon receiving a positive result within a very limited time. It is normal for all pregnant women to wish for a healthy child, which motivates them to take the NIPT to be “relieved [27].” However, even when they are relieved upon receiving negative results, many women were still ambivalent about their decision, feeling guilty about having tried to decide their child’s life, or having felt denial towards individuals with disabilities, but also having to endorse the thoughts about the test. This is probably what made them recognize their ambivalence and internal conflict at having these unresolved complex emotions.

## Conclusion

The present study shows that even negative results can induce ambivalent feelings among pregnant women, and it is important to recognize that these feelings may be due to interrelated factors concerning genetic counseling, environmental factors, and increased anticipatory anxiety. Moreover, pregnant women have requested additional information regarding the detailed prognosis of individuals with Down syndrome or other congenital disorders and/or artificial abortion, assuming positive NIPT results. Therefore, genetic counseling and appropriate follow-up sessions are essential for the well-being of pregnant women taking the NIPT.

### Limitation and future direction

There may be some possible limitations to this study. Firstly, a possible bias due to only women filling in the free description column of the questionnaire after 1 year; the background of these women is also not clear. Further prospective studies are needed to investigate the psychosocial situations of women undergoing NIPT before and after the examination and after the passage of time. Even in the case of a negative NIPT result, women may have various conflicts and ambivalent feelings, and recognizing the factors that elicit these feelings may help future genetic counseling.

### Practice implications

To reduce internal conflict in pregnant women due to ambivalent feelings, it is critical to actively provide them detailed information about living with a child affected by one of the disorders screened for during the genetic counseling sessions and to discuss this information, assuming a potentially positive result, including specifics regarding medical pregnancy termination. Importantly, we believe that pregnant women should receive personalized psychological care so that they can make independent decisions based on factual knowledge. Thus, even if pregnant women experience feelings of ambivalence, they should be reassured and accept that undergoing NIPT was their decision after careful consideration, thereby reducing their potential internal conflicts.

## Data Availability

The datasets used and/or analysed during the current study are available from the corresponding author on reasonable request.

## Abbreviation

NIPT: Non-invasive prenatal testing

## References

[1] Nakic Rados S, Kosec V, Gall V (2013). The psychological effects of prenatal diagnostic procedures: maternalanxiety before and after invasive and noninvasive procedures. Prenat Diagn.

[2] Richmond Z, Fleischer R, Chopra M, Pinner J, D'Souza M, Fridgant Y, Hyett J (2017). The impact of non-invasive prenatal testing on anxiety in women considered at high or low risk for aneuploidy after combined first trimester screening. Prenat Diagn.

[3] van Schendel RV, Page-Christiaens GC, Beulen L, Bilardo CM, de Boer MA, Coumans AB, Faas BH, van Langen IM, Lichtenbelt KD, van Maarle MC (2016). Trial by Dutch laboratories for evaluation of non-invasive prenatal testing. Part II-women's perspectives. Prenat Diagn.

[4] Lo TK, Chan KY, Kan AS, So PL, Kong CW, Mak SL, Lee CN (2019). Decision outcomes in women offered noninvasive prenatal test (NIPT) for positive down screening results. J Matern Fetal Neonatal Med.

[5] Garcia E, Timmermans DR, van Leeuwen E (2008). Rethinking autonomy in the context of prenatal screening decision-making. Prenat Diagn.

[6] Koletzko SH, La Marca-Ghaemmaghami P, Brandstatter V (2015). Mixed expectations: effects of goal ambivalence during pregnancy on maternal well-being, stress, and coping. Appl Psychol Health Well Being.

[7] Jallinoja P, Hakonen A, Aro AR, Niemela P, Hietala M, Lonnqvist J, Peltonen L, Aula P (1998). Attitudes towards genetic testing: analysis of contradictions. Soc Sci Med.

[8] Dormandy E, Michie S, Hooper R, Marteau TM (2006). Informed choice in antenatal Down syndrome screening: a cluster-randomised trial of combined versus separate visit testing. Patient Educ Couns.

[9] Lewis C, Hill M, Chitty LS (2016). A qualitative study looking at informed choice in the context of non-invasive prenatal testing for aneuploidy. Prenat Diagn.

[10] Conner M, Povey R, Sparks P, James R, Shepherd R (2003). Moderating role of attitudinal ambivalence within the theory of planned behaviour. Br J Soc Psychol.

[11] Yotsumoto J, Sekizawa A, Suzumori N, Yamada T, Samura O, Nishiyama M, Miura K, Sawai H, Murotsuki J, Kitagawa M (2016). A survey on awareness of genetic counseling for non-invasive prenatal testing: the first year experience in Japan. J Hum Genet.

[12] Samura O, Sekizawa A, Suzumori N, Sasaki A, Wada S, Hamanoue H, Hirahara F, Sawai H, Nakamura H, Yamada T (2017). Current status of non-invasive prenatal testing in Japan. J Obstet Gynaecol Res.

[13] Reid B, Sinclair M, Barr O, Dobbs F, Crealey G (2009). A meta-synthesis of pregnant women's decision-making processes with regard to antenatal screening for Down syndrome. Soc Sci Med.

[14] Hurford E, Hawkins A, Hudgins L, Taylor J (2013). The decision to continue a pregnancy affected by Down syndrome: timing of decision and satisfaction with receiving a prenatal diagnosis. J Genet Couns.

[15] Reed AR, Berrier KL (2016). A qualitative study of factors influencing decision-making after prenatal diagnosis of Down syndrome. J Genet Couns.

[16] van Schendel RV, Page-Christiaens G, Beulen L, Bilardo CM, de Boer MA, Coumans ABC, Faas BHW, van Langen IM, Lichtenbelt KD, van Maarle MC (2017). Women’s experience with non-invasive prenatal testing and emotional well-being and satisfaction after test-results. J Genet Couns.

[17] Williams C, Alderson P, Farsides B (2002). Is nondirectiveness possible within the context of antenatal screening and testing?. Soc Sci Med.

[18] Perry CL, Henry MJ (2010). Exploring adoption with clients: the need for adoption education within the genetic counseling profession. J Genet Couns.

[19] Allison SJ, Stafford J, Anumba DO (2011). The effect of stress and anxiety associated with maternal prenatal diagnosis on feto-maternal attachment. BMC Womens Health.

[20] McMahon CA, Ungerer JA, Beaurepaire J, Tennant C, Saunders D (1997). Anxiety during pregnancy and fetal attachment after in-vitro fertilization conception. Hum Reprod.

[21] Fisher J, Wynter K, Hammarberg K, McBain J, Gibson F, Boivin J, McMahon C (2013). Age, mode of conception, health service use and pregnancy health: a prospective cohort study of Australian women. BMC Pregnancy Childbirth.

[22] Hjelmstedt A, Widstrom AM, Collins A (2006). Psychological correlates of prenatal attachment in women who conceived after in vitro fertilization and women who conceived naturally. Birth.

[23] Tendais I, Figueiredo B (2016). Parents’ anxiety and depression symptoms after successful infertility treatment and spontaneous conception: does singleton/twin pregnancy matter?. Hum Reprod.

[24] Kleinveld JH, Timmermans DR, de Smit DJ, Ader HJ, van der Wal G, ten Kate LP (2006). Does prenatal screening influence anxiety levels of pregnant women? A longitudinal randomised controlled trial. Prenat Diagn.

[25] van den Berg M, Timmermans DR, ten Kate LP, van Vugt JM, van der Wal G (2006). Informed decision making in the context of prenatal screening. Patient Educ Couns.

[26] Richards EG, Sangi-Haghpeykar H, McGuire AL, Van den Veyver IB, Fruhman G (2015). Pregnant patients’ risk perception of prenatal test results with uncertain fetal clinical significance: ultrasound versus advanced genetic testing. Prenat Diagn.

[27] Sapp JC, Hull SC, Duffer S, Zornetzer S, Sutton E, Marteau TM, Biesecker BB (2010). Ambivalence toward undergoing invasive prenatal testing: an exploration of its origins. Prenat Diagn.

